# Investigating the performance of multivariate LSTM models to predict the occurrence of Distributed Denial of Service (DDoS) attack

**DOI:** 10.1371/journal.pone.0313930

**Published:** 2025-01-17

**Authors:** Prashant Kumar, Chitra Kushwaha, Dimple Sethi, Debjani Ghosh, Punit Gupta, Ankit Vidyarthi

**Affiliations:** 1 Bennett University (The Times Group), Greater Noida, Uttar Pradesh, India; 2 DRDO-Institute for System Studies & Analyses, Civil Lines, Delhi, India; 3 University College Dublin, Dublin, Ireland; 4 Pandit Deendayal Energy University, Gandhinagar, India; 5 Jaypee Institute of Information Technology, Noida, India; ICFAI Foundation for Higher Education Faculty of Science and Technology, INDIA

## Abstract

In the current cybersecurity landscape, Distributed Denial of Service (DDoS) attacks have become a prevalent form of cybercrime. These attacks are relatively easy to execute but can cause significant disruption and damage to targeted systems and networks. Generally, attackers perform it to make reprisal but sometimes this issue can be authentic also. In this paper basically conversed about some deep learning models that will hand over a descent accuracy in prediction of DDoS attacks. This study evaluates various models, including Vanilla LSTM, Stacked LSTM, Deep Neural Networks (DNN), and other machine learning models such as Random Forest, AdaBoost, and Gaussian Naive Bayes to determine the DDoS attack along with comparing these approaches as well as perceiving which one is about to give elegant outcomes in prediction. The rationale for selecting Long Short-Term Memory (LSTM) networks for evaluation in our study is based on their proven effectiveness in modeling sequential and time-series data, which are inherent characteristics of network traffic and cybersecurity data. Here, a benchmark dataset named CICDDoS2019 is used that contains 88 features from which a handful (22) convenient features are extracted further deep learning models are applied. The result that is acquired here is significantly better than available techniques those are attainable in this context by using Machine Learning models, data mining techniques and some IOT based approaches. It’s not possible to completely avoid your server from these threats but by applying discussed techniques in the present juncture, these attacks can be prevented to an extent and it will also help to server to fulfil the genuine requests instead of sticking in the accomplishing the requests created by the unauthentic user.

## I. Introduction

These days, denial-of-service (DoS) attacks are quite common. Denial of Service attacks occur when a server receives more requests than it can handle and refuses to process them. This type of attack occurs when the server is overloaded with network traffic, which can be both authorized and unauthorized. Distributed Denial of Service (DDoS) attacks are similar to DoS attacks, but DDoS attacks are carried out by multiple compromised devices, often from various locations, to overwhelm a target system [1]. First time the DDoS attack came into picture in 1996 when one of the oldest internet service providers, Panix had to be offline for many days. There are several types of DDoS attack but mainly three types of DDoS attacks are: Application Layer Attack, Protocol attack, Volumetric attacks [2]. All the three debilitate the resources by their distinct techniques by targeting the different layers of OSI model. While there are number of techniques present to avoid in which some are general and some are technology based. DDoS attack can be prevented to an extent by setting a continuous monitoring, limiting network broadcasting, ensuring server redundancy, improving network security (phonixnap.com). But these general techniques are not much effective so Technology based some prediction procedure came into picture those are basically machine learning based and some are also using IOT based approach. But these techniques are not so powerful for much accurate results because of machine learning model limitations (as machine learning cannot perform efficiently with large dataset) [3]. So, in this paper, we are practicing with deep learning algorithms to implement the model, since deep learning models are considerably better than machine learning models.

**Fig 1:**
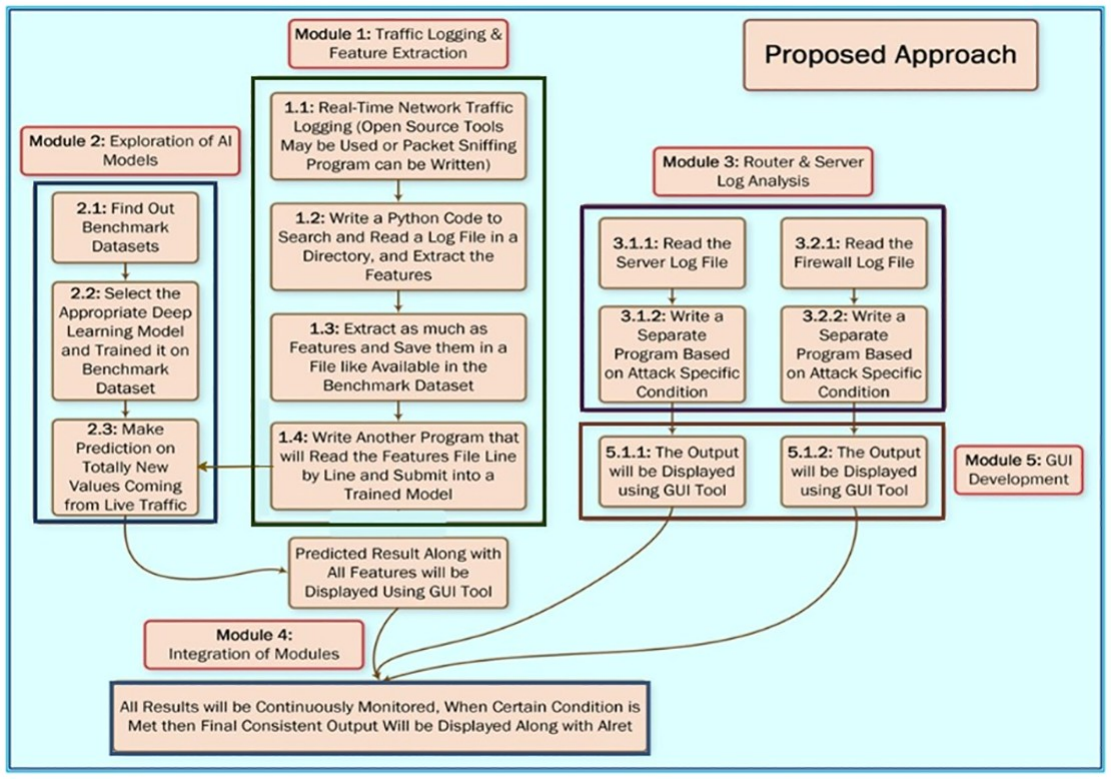
Proposed framework for Distributed Denial of Service (DDoS) attack detection using deep learning.

So here we are targeting to predict the DDoS attack by observing the deviations occurred at the environment of the server when the incursion is about to be, so that we can inhibit it by Deep learning models Vanilla-LSTM, Stacked-LSTM, ANN [3–5]. It’s not like that up to now no one has operated on this, some has worked on LSTM and RNN to restrain and disposed liable precision but still lacking in furnishing systematic outcomes, so to fulfilling this gap the technique we are applying here is performed on the CICDDoS2019 dataset with some auxiliary models. Fig 1 depicts the whole strategy. Moreover, the selection of LSTM networks is based on their superior ability to handle sequential data with temporal dependencies, their robustness against common RNN limitations, and their successful application in similar cybersecurity contexts. This makes them a fitting choice for evaluating the detection of DDoS attacks and ensuring the reliability and accuracy of the model in real-world scenarios.

The implementation that is used here contains the following contributions:

- To select an appropriate benchmark dataset and perform the preprocessing on dataset
- To design and develop the model using Long Short-Term Memory (Vanilla LSTM, Stacked LSTM) that remembers the features value of required neural networks.
- To design and develop the model using Artificial Neural Network, AdaBoost, GaussianNB and Random Forest.
- To design and develop an interactive user interface that enables Real-Time DDoS Detection.
- To obtain the comparison report of accuracy of all the six models.

This paper contains 5 sections: Section 1 discusses the introduction part, Section 2^nd^ details about the Literature Survey, Section 3^rd^ describes the methodology, Section 4^th^ and 5^th^ reveals the algorithm and results obtained in the paper respectively, and the last section 6^th^ details conclusion of this paper.

## II. Related work

There are discussed some scholarly articles those are written till now in reference to solve the DDoS attack problem. Described below methods are based on three approaches: General methods, Machine learning based approaches, Deep Learning based approaches.

### A. General methods

In 2020, [1] focused on Named Data Networking to detect and mitigate DDoS attacks by cooperating between Named Data Networking routers, which hold data structures such as Forwarding Information Base (FIB), content store (CS), and Pending Interest Table (PIT), and detecting the DDoS attack with the help of a centralized controller. They have improved the effectiveness and precision of detecting bogus name prefixes when compared to the previously presented method. Moreover, in 2019, [2] authors introduced a technique to identify HTTP flooding attacks using the Susceptible Infective Susceptible (SIS) mathematical model, which is capable of measuring many sorts of user parameters and detecting anomalous user behavior.

### B. Machine learning based approaches

General methods were not able to give efficient results in long term so there was a need to give an approach which can be beneficial in long term also. So, researchers started to work on Machine learning based approaches. In 2022, [3] proposed a machine learning solution by radial basis function with cuckoo search algorithm to detect application layer DDoS attack by using Generic algorithm that finds optimal features, those are used to trained in cuckoo search with radial basis function neural network. They compared their technologies with well-known methods as well as finds that their proposed method is able to give lower rate. Till now whatever the technology has been discussed, working efficiently but there was the absence of time-based features so in 2021 [4] uses 25 time-based features to detect DDoS as well as binary and multiclass classification technique to classify DDoS attack types based on multiclass classification. In 2021, [5] has proposed a method to detect DDoS attack using Artificial Neural Network that detects all the application layer DDoS attack (HTTP flood, UDP flood, Smurf and SiDDoS), Network and transport layer DDoS attack as well as calculating the Time and space complexity to improve the efficiency along with perceiving significantly better results. In 2021, [6] has discussed different DDoS defense system based on ML techniques in virtualized network, cloud computing and software defined network, IOT environment and suggests number of directions for research. In 2020, [7] have proposed a model to detect and mitigate the DDoS attack by using reinforcement learning. Basically, they have advanced the state-of-the-art techniques by using two agents on per flow basis for any type of network environment and showed a considerable increase in good put of legitimate TCP traffic for many choices of host density. In 2020, [8] has focused on DDoS attack detection in software defined network by using factorization machine based to extract the features and has achieved 95.80% accuracy along with this given the defense method that has gained 97.85% success rate. In 2020, [9] has proposed a solution to control suspicious activities by using machine learning algorithm as well as calculating efficiency of mode by two methods: firstly, by feature elimination method (Recursive feature selection method) and secondly by considering those features which are concerned in recent works based on the DDoS detection and gained success. In 2020, [10] used to work on the basis of that better results depends on dataset and KDD-CUP dataset has been used by them to perform K-NN, ID3, Naïve Bayes and C4.5 algorithms to compare all obtained results those are experimentally verified. In 2020, [11] proposed two methods first to identify a DDoS attack and second to discover the DDoS using improved K-NN based on machine learning in software defined network and given a theoretical as well as experimental analysis along with this showed that proposed method detects better in comparison of other methods.

In 2020, [12] proposed an advanced technique of state-of-the-art method, in which they detect the botnets which are used by the attackers to perform DDoS attack along with this also provide unsupervised statistical learning approach to analyze the traffic based on botnets on CAIDA, Botnet 2014 dataset. In 2019, [13] Static classification algorithm (Naive Bayes, Decision Tree (Entropy), Decision Tree (Gini), and Random Forest) were used to detect dynamic DDoS attack and prepared a detection system that is the combination of distributed system and fuzzy logic system that dynamically selects classification algorithm and observes that fuzzy logic system can efficiently perform in this. In 2019, [14] has first given the review on the existing dataset and secondly proposed a classification-based approach based on set of network flow features and performed the experiment on own generated dataset named CICDDoS2019 which is going to be used to in this paper also. In 2019, [15] has discussed methods for detecting and mitigating attack by the Robust Principal Component Analysis to find correlation and dependencies in computer access patterns and presents a model that is able to provide efficient results. In 2018, [16] have used the Hadoop architecture to increase the speed of processing requests on large files and given a method based on Time series analysis to blocking suspicious IP address and prevent the malicious users to accessing and it was able to provide the suitability to detect DDoS in the duration of five minutes and act accordingly.

### C. Deep learning based approaches

As conventional ML based techniques cannot extract high performance features automatically so Deep learning-based approaches came into picture that gives efficient results in comparison to machine learning methods. In 2022, authors [17] suggested a strategy that involved analyzing the volume of live traffic in a resource-constrained setting. They employed a light weighted deep learning model on the most recent dataset and demonstrated that their proposed methodology is acceptable for DDoS detection and has the same accuracy as the existing state of the art solutions. In 2021, [18] discussed a hybrid solution called AE-MLP, which is essentially a combination of Auto Encoder (used for effective feature selection) and multilayer perceptron Network (used to categorized the selected features into DDoS kinds), and obtained 98% accuracy on the CICDDoS2019 dataset. In 2021, [19] has proposed a model to detect application layer and transport layer DDoS attack in software defined architecture by using machine learning and deep learning model and performed the experimental analysis on CICDDoS2017 and CIC2019DDoS2019 dataset and by applying suitable Support Vector Machine (SVM), K-nearest neighbor (K-NN), multilayer perceptron (MLP), convolutional neural network (CNN), gated recurrent units (GRU) and achieved the 98% accuracy for transport layer and 95% accuracy for application layer DDoS attack. In 2021, [20] presented a study on that how the Deep learning approaches are far better than state of the art approaches. They had given review and analysis based on previous works on the DDoS attack in software defined network and suggested an overall system architecture. In 2020, [21] proposed a methodology that uses deep convolutional neural network to detect the DDoS attack in Software Defined Network and the This framework is evaluated on a current state-of-the-art Flow-based dataset under established benchmarks. Improved accuracy is demonstrated against existing related detection approaches. In 2018, [22] had worked on Open-flow based Software defined network environment and given a deep learning model that consists input layer, forward recursive layer, reverse recursive layer, fully connected hidden layer and output layer to detect DDoS attack this model was able to give considerably better results than conventional machine learning ways. In a few research, LSTM models were also used, such as in article [23], where the authors proposed an LSTM (Long Short-Term Memory) model to detect DDoS threats by analyzing network data. The paper was published recently in 2023. However, they did not address the variants of LSTM models and did not provide a comprehensive system with a GUI for deployment in real-time applications.

After analyzing all above, one thing is observed that performance of Deep learning models is considerably better but still there is a need of improvement in context of remembering the features to increase the accuracy of the model. Moreover, it’s not as if no one has worked on this before; some have used LSTM and RNN to constrain and dispose of liable precision, but they still fall short of providing systematic results. So, in this paper, we have used LSTM, ANN and Random Forest models those provides maximum accuracy on latest dataset.

## III. Data preporcessing

**Fig 2:**
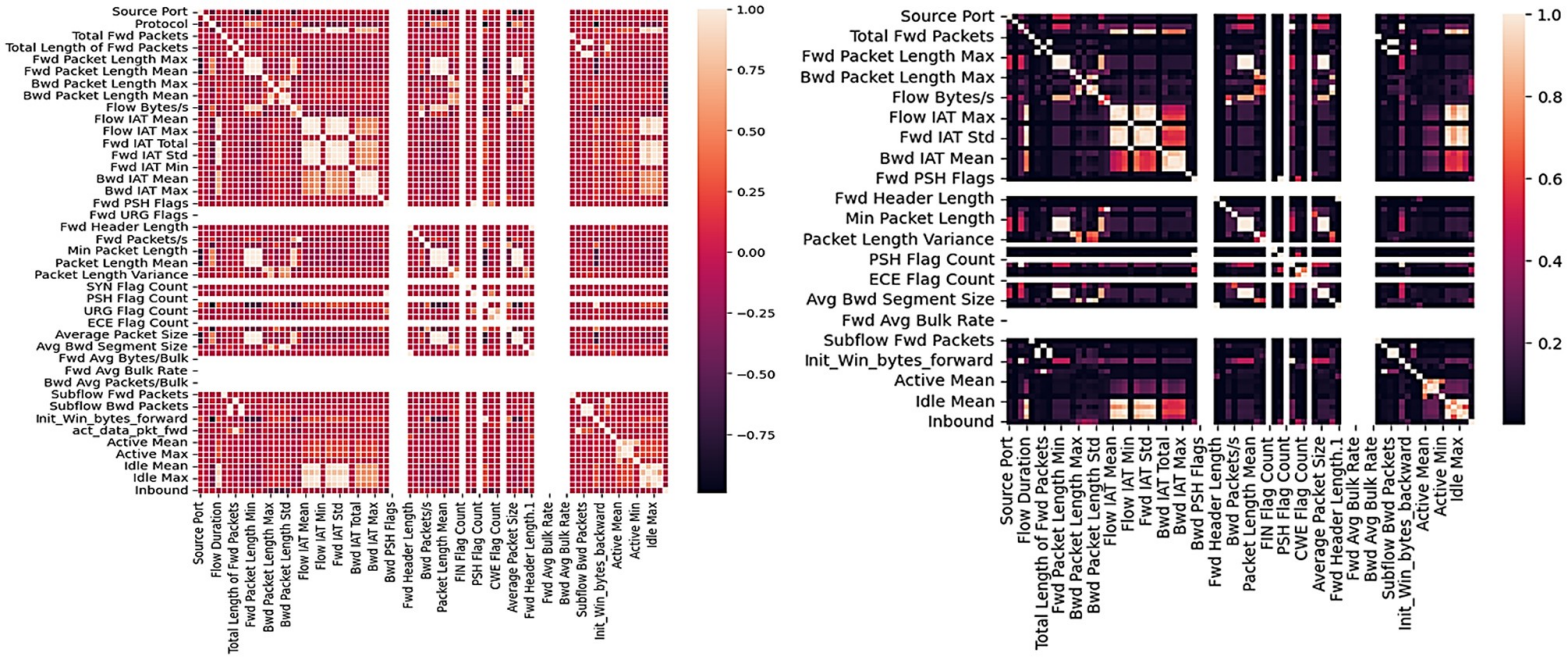
Correlation matrix to check whether features are positive/negative correlated with one another.

**Fig 3:**
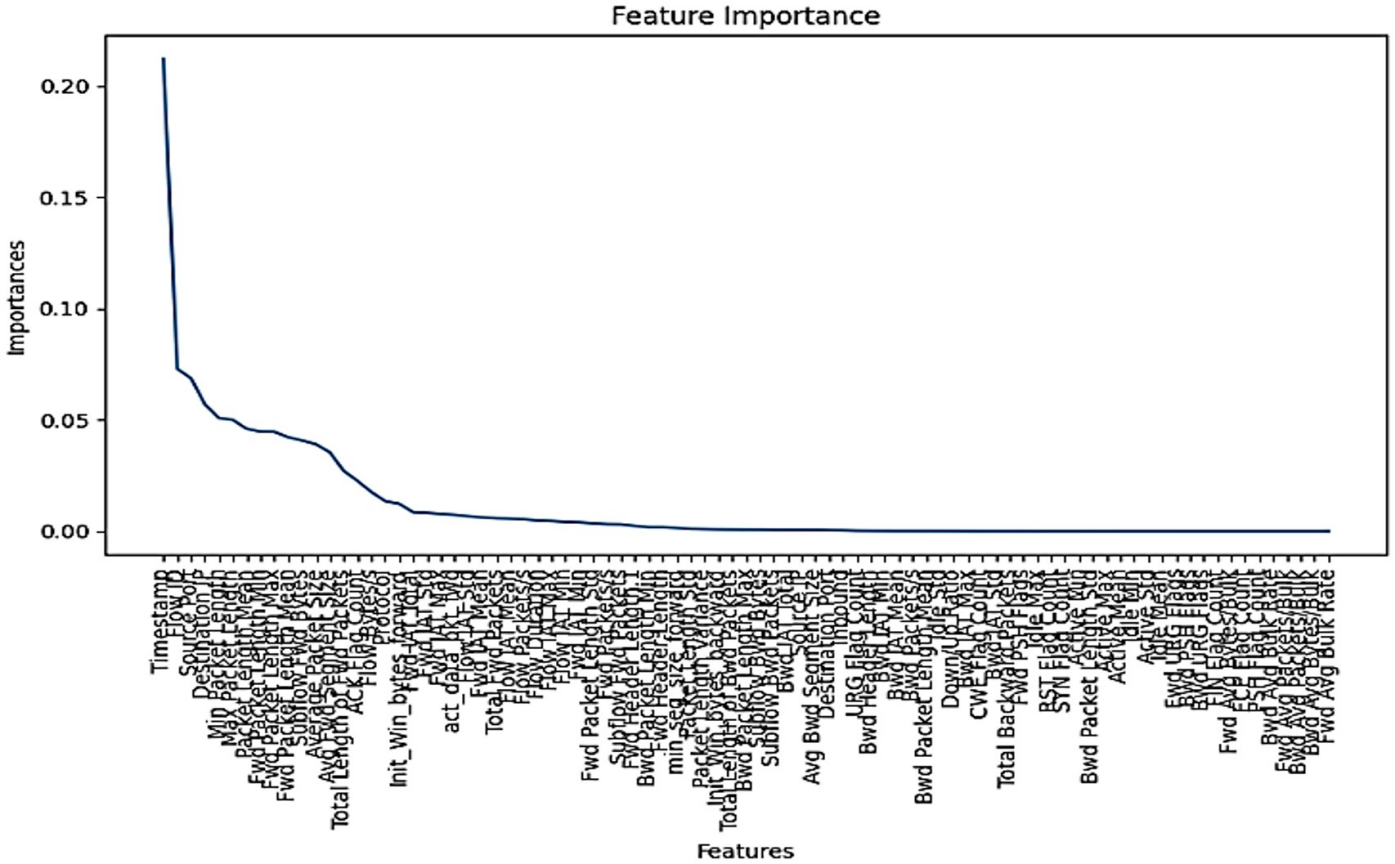
Features selection by Random Forest.

**Fig 4:**
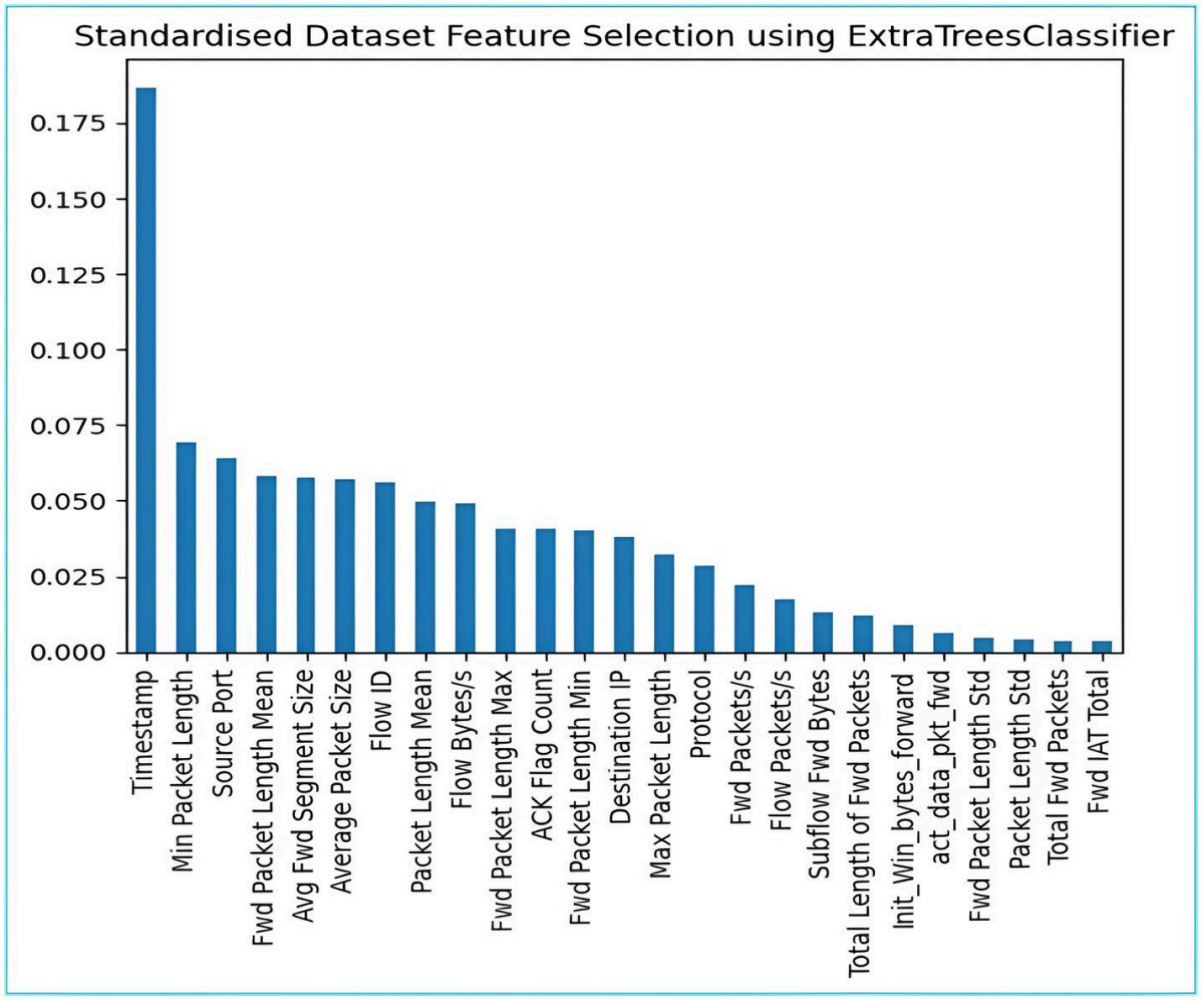
Features selection by ExtraTreesClassifier.

In the first step towards implementing DDoS attack using deep learning preprocessing of the dataset is an important step. In this, we perform checking the relevance of the features, cleaning in our data, and remove irrelevant information from the dataset (Figs 2–4), this preprocessing of the dataset does not only save computational time as well as help us to achieve result that contains maximum accuracy. Firstly, we have selected the CICDDoS2019 dataset because latest one and free from any type of limitations. It is one of the most comprehensive and well-structured datasets available for DDoS attack detection. It provides a diverse range of attack scenarios and realistic traffic patterns that closely resemble modern network environments, making it suitable for evaluating the performance of various detection models. This dataset contains 9327647 number of rows and 88 number of columns (Table 1). In this dataset all rows and columns are not providing the relevant information so we have to identify and remove this type of value and select only those features which are providing most relevant information about the data. In these stage two steps are to be performed:

1. Feature Engineering
2. Feature Selection

**Table 1:** Dataset classes along with number of samples.

S. No.	Types of Attacks Included in the Dataset	No of Samples Before Pre- processing	No of Samples After Pre- processing	Training Samples	Testing Samples
1	MSSQL	1736241	1675417	6332454	2713909
2	DrDoS_SNMP	1547996	1544765		
3	DrDoS_DNS	1521333	1472536		
4	Syn	1285501	1200336		
5	UDP	1126407	1103731		
6	NetBIOS	1097110	1054471		
7	LDAP	574613	561813		
8	DrDoS_NTP	360786	358745		
9	Portmap	56072	53200		
10	BENIGN	21588	21349		
	**Total**	**9327647**	**9046363**	**6332454**	**2713909**

To preprocess the data, strongly correlated features are first analyzed using a correlation matrix since they might have a negative impact on the performance of some models, such as linear regression, by introducing redundancy and making it more difficult to isolate the effect of each individual feature. A correlation matrix can help you locate strongly linked pairs of features, allowing you to remove or combine those features to reduce redundancy. Following that, feature engineering and feature selection approaches are applied.

Feature Engineering (Handling Missing Values): In real world dataset there is a problem of missing value and it’s very common but if it is present in dataset, it can decrease the accuracy of model so it is very important to deal with this. There are some methods by following which missing values can be removed one other case is when the metadata of any column is missing in that case, we can remove that column also. But in our dataset, there are 281284 number of missing values are present so we have removed those rows but all the class labels are present so no need to ignore any tuple. Another method to handle these values is to fill these values manually that can consume much time in case of large dataset so it is not preferred. To fill these missing values, we can use central tendency values it means if data is normally distributed then these missing values are filled by mean and if data is skewed distributed then median is used to fill these missing values. We can also replace with the most frequent values of that column. Encoding: In this step we have to observe every column value means which type of value it contains. Algorithms work with only numerical type of values if any tuple contains categorical value or not a number (NaN) type of value then it should be converted into numerical value because after that only machine learning and deep learning models can be applied on the dataset. There are mainly two types of encoding techniques: Nominal Encoding and Ordinal Encoding.

In Nominal Encoding we assign the integer values to the labels without worrying about the ranking. In this integer values are not treated according to the priorities. To perform this there are three techniques: Mean Encoding, One Hot encoding, and One Hot Encoding with multi categorical values. In mean encoding, label is replaced with the mean value of target in One Hot Encoding technique, while in one hot encoding, we split that feature and create as many numbers of columns as categorical values are present in that feature and place there 0 or 1 but if there are a greater number of categorical value so it will lead to increase the number of features unnecessarily. To overcome this problem multiple integer values are assigned for each different label for multi categorical values. In Ordinal encoding, we have to consider the ranking also while assigning the integer to labels basically two methods are there to perform this: Label encoding and Target guided Ordinal encoding. Different Integer values are assigned to different labels smaller number contains the lowest priority although greater number contains highest priority. While in Target Guided Ordinal encoding mean is calculated in respect of target variable and according to the mean values ranking of label is decided by integer value. In the encoding of our dataset first we have found out the number of features those are having categorical values then applied label encoding. This Label encoder converts non numerical values into numeric values.

Data Transformation: To restrict the range of any features value feature transformation is used. To perform this there are mainly two methods: Normalization and Standardization. To standardize the data, we have used standard scaler in our dataset. In this first we subtract the feature by mean value, find standard deviation and divide every features value by it.

Feature Selection (Extra Tree Classifier/Random Forest): In every dataset there are many numbers of features but all features are not relevant, to identify those irrelevant features, feature selection method is used. In our methodology, we have used Extra tree classifier that constructs decision tree by transformed data (obtained after performing previous step) and then at every test node randomly a sample of m out of n features is assigned to this constructed decision tree and then calculated feature importance for every tuple, selected 22 best features out of 85 features based on the value of Gini importance (Figs 2–4).

## IV. Algorithm

LSTM (Long Short-Term Memory): We have selected LSTM model because we have to predict the values in every period of time and in this context LSTM works best. In this, each features value does not depend only on its past value but also has some dependencies on other previous values. LSTM is the extended version of RNN because only memory layer is added into hidden layer and RNN is not able to remember previous outputs but LSTM has this capability. It consists of three gates: Input gate, forget gate and output gate.

Whole working of this model depends mostly on the forget gate this basically decides how much previous data is to be remember and for how much time. it uses sigmoid function that is nonlinear activation function, maintains the value between 0 to 1 and helps in updating or forgetting the value. If its value is 0 it means information is being forgotten. This sigmoid function (*ft*) is calculated by passing the information from current state (*x*_*t*_) and previous hidden state (*h*_*t-1*_). The input gate (*i*_*t*_) determines how much input of the current moment need to be saved for further requirements. It first passes the information of current state (*x*_*t*_) and hidden state (*h*_*t-1*_) to second sigmoid function as well as to tanh function. Now these both values (obtained from Sigmoid function [between 0 to 1] and tanh [between -1 to 1] function) will be applied in point-by-point multiplication. These two gates basically control the state of the memory cell. The previous cell state (*C*_*t-1*_) and output of forget gate (*f*_*t*_) is multiplied and in between of this outcome and output of input gate (*i*_*t*_) point to point addition is performed. The output gate calculates the value for next hidden state by passing the value of current state and previous hidden state into sigmoid function and cell state information is passed through tanh function then both the values (sigmoid function and tanh function) pass by point-to-point multiplication.


$$f_{t}=sigmoid(W_{f}*[h_{t-1},x_{t}]+b_{f})(Output\;of\;Forget\;gate)$$

$$i_{t}=sigmoid(W_{i}*[h_{t-1},x_{t}]+b_{i})(Output\;of\;Input\;gate)$$

$${\tilde{S}}_{t}=tanh(W_{c}*[h_{t-1},x_{t}]+b_{c})$$

$${\tilde{C}}_{t}=f_{t}*(C_{t-1})+i_{t}*{\tilde{C}}_{t}(Output\;of\;State\;cell)$$

$$o_{t}=sigmoid(W_{o}*[h_{t-1},x_{t}]+b_{o})$$

LSTM model have mainly four variants named: Univariate LSTM models, Multivariate LSTM models, Multistep LSTM model and Multivariate Multistep LSTM model. In Univariate LSTM model only one hidden layer and one output layer is used for prediction. While in Multivariate LSTM model multiple variables are present to affect the outcome. Though in dataset CICDDoS2019, there are nonlinear interdependencies so we have used multivariate LSTM model to train our dataset. In this each features value does not depends only on its past value but also has some dependencies on other previous values. In sequence of applying Multivariate LSTM model, multivariate input data is used in multivariate input data, data can be of two types one is stationary data and other one is non-stationary data. If mean, variance and covariance are constant with time then it is said to be stationary data while if these values are varying with time, then it is said to be nonstationary data. So first we have checked that our data is stationary or non-stationary by visual test and by statistical test. Visual test is not fully accurate so for surety we use statically test in this there are two methods.

1) Augmented Dickey Fuller and
2) Kwiatkowski-Phillips Schmidt-Shin (KPSS)

In the dataset that we have used, non-stationary data is present so first we have to convert it into stationary (because Multivariate LSTM model is time series forecasting data) data then only we can apply multivariate LSTM model so use seasonal differencing method:

$${\text{Y}}_{\text{t}}={\text{Y}}_{\text{t}}-{\text{Y}}_{\text{t}-\text{n}}$$

There are two more methods to convert it in stationary data differencing (Y_t_ = Y_t_ − Y_t-1_) and transformation but in our model, we have used seasonal differencing.

## V. Methodology

The entire approach is shown in the Fig 1. For the better understanding, it is divided into 5 modules: Traffic logging and feature extraction, Exploration of AI models, Router and server log analysis, Integration of modules, and GUI Development. All the modules are covered briefly in the following subsections:

### A. Traffic logging and feature extraction

Initially, network traffic is captured using an open-source tool as well as a self-written packet sniffing program in Python. An open-source tool called Wireshark is installed and configured on a system connected to the network. Soon after, traffic is captured and saved into a .pcap file. Thereafter, features are extracted from the .pcap file using the CICflowmeter of the University of New Brunswick. Its real-time traffic ultimately has to be fed to a trained deep learning model to make a prediction of a DDoS attack. Therefore, the extracted features must be the same as those available in some benchmark datasets because a deep learning model has to be trained on them. Therefore, a total of 88 features are extracted from real-time network traffic using CICflowmeter, and the same features are also available with the CICDDoS2019 dataset. Out of them, the selected best features (22) are chosen and saved in .csv file, and a Python program is written that can locate the log file (.csv) in a directory, read the best selected features (22) row by row, and feed them into a trained deep learning model.

### B. Exploration of AI model

This module is parallel to the first module, in which all available benchmark datasets are explored. They are checked on the basis of the number of DDoS-related features included in the dataset as well as their recentness. The citation of the dataset is also taken into account. Ultimately, a CICDDoS2019 dataset is selected for this research work. Once the dataset is finalized, a deep learning model called LSTM is selected for this research work based on the classification accuracy obtained on different types of applications recently. In this research, two variants of LSTM, called Vanila LSTM and Stacked LSTM, are used. These variants are trained, validated, and tested on the selective features of the previously selected dataset, i.e., CICDDoS2019. Thereafter, the selected features extracted from the real-time network traffic in the previous module are fed into these trained deep learning models, and predicted results are displayed using the GUI tool.

### C. Router and server log analysis

A separate Python program is also written to analyze the server and firewall logs to find traces of the DDoS attack. The program was written to analyze the following scenarios: how many requests are being made from the same machine, how many systems are involved in the same types of requests, and how frequently these systems are generating these requests.

### D. Integration of modules

This module is all about ensuring reliable information flow between all modules whenever it is required. For example, module one output (extracted features) needs to be fed into the trained models of module one. It is possible only when the required features match the supplied features. This compatibility is ensured by this module. Also, these modules collaborate with the GUI Development module so that all the features, along with predicted outcomes, could be displayed in the GUI tool.

### E. Gui development

Ultimately, this module provides an interactive GUI tool (Fig 7) that assists users in easily deploying DDoS detection mechanisms into any network they want to secure. The given tool is rich in functionality; it is not just providing predicted results and extracted features. This tool provides the following functionalities: General functionality, traffic capture, dataset-related operations, training and testing of deep learning models, real-time DDoS prediction, writing the snort rule and calling snort, etc. General functionality covers basic network-related commands such as ping, netstat, tracert, hostname, ipconfig, ipconfig/all, nslookup, etc. Under the traffic capturing title, various tools are embedded in our GUI that can be invoked just by clicking the corresponding buttons, such as Snort, Wireshark, CICflowmeter, TCPdump, etc. Moreover, dataset-related operations include bifurcation of the dataset for training, validation, and testing, visualization of the dataset, feature selection, etc. Apart from the above-mentioned operations, this tool also assists in hyperparameter setting for training and conducting testing on a trained model. We can also save the trained model if required. Next time, whenever you need to make a prediction, there is no need to train the model again. The earlier saved model can be loaded into memory, and new values are fed to it to make a real-time prediction. It displays extracted feature values line by line along with predicted results in almost real-time. Eventually, you can also write snort rules and deploy them as and when required for ddos detection.

### F. Implementation of LSTM models

**Fig 5:**
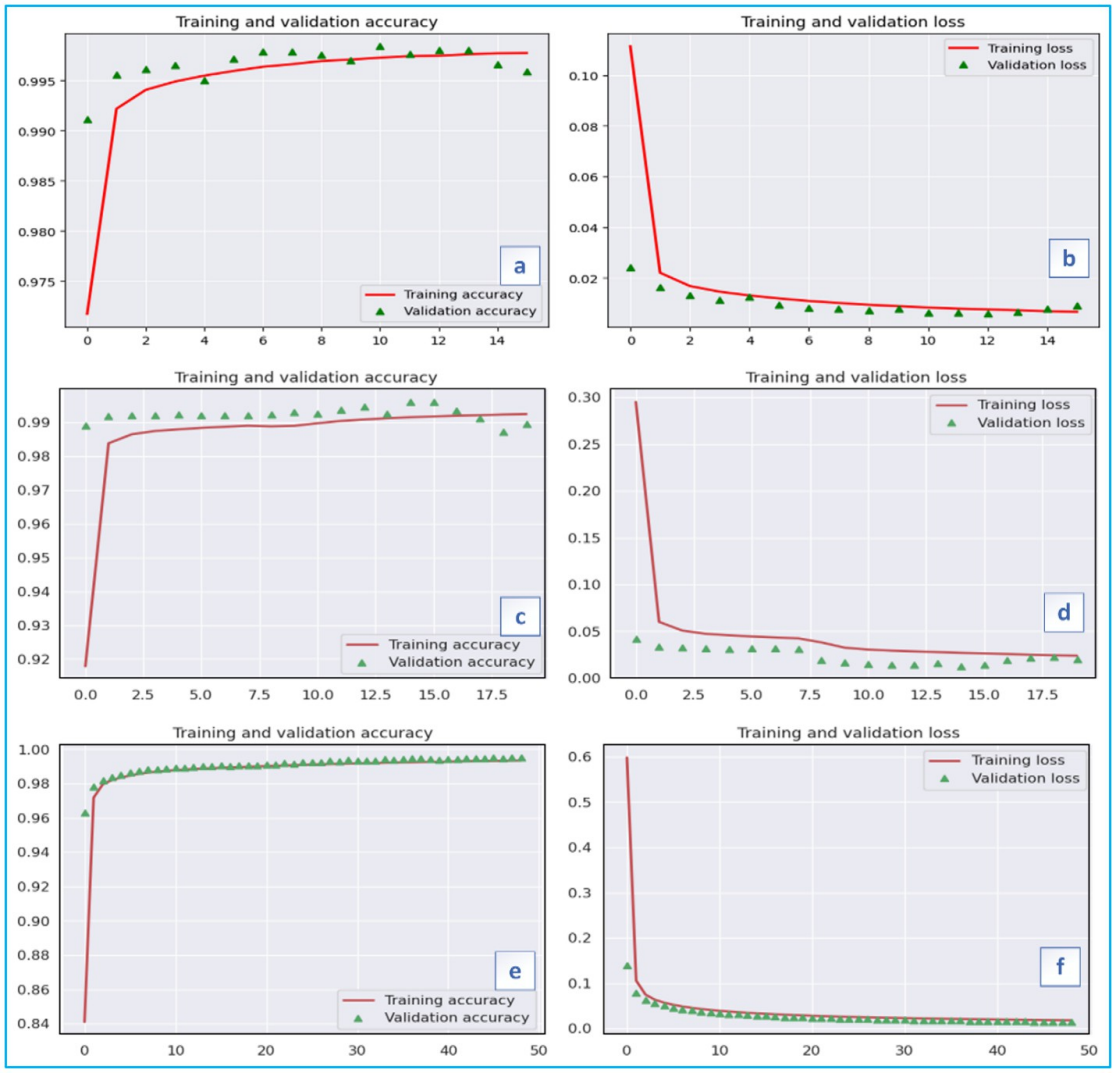
Training Results Demonstrate the Accuracy and Loss for Various Models: (a, b) Vanilla LSTM, (c, d) Stacked LSTM, (e, f) Deep Neural Network.

**Fig 6:**
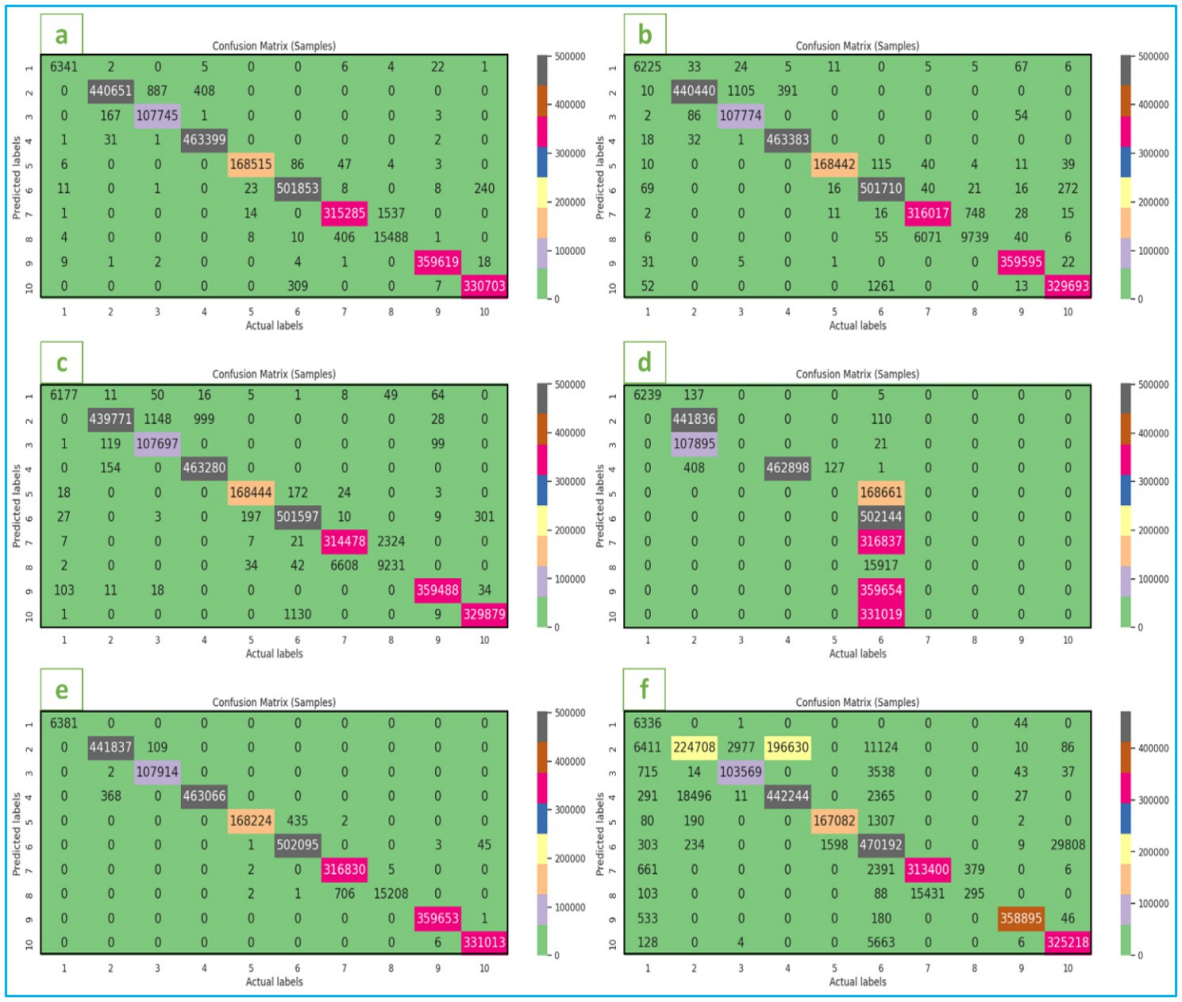
Confusion Matrix: (a) Vanilla LSTM, (b) Stacked LSTM, (c) Deep Neural Network, (d) AdaBoost, (e) Random Forest, (f) GaussianNB.

This work considers two multivariate LSTM models (Vanila LSTM and Stacked LSTM). These models’ performance is also compared to that of other competitive models, including Deep Neural Network, AdaBoost, Random Forest, and GaussianNB. The vanilla LSTM is built with a single hidden layer that contains LSTM units made up of an input gate, a forget gate, an output gate, and a cell, whereas the stacked LSTM is built with multiple hidden LSTM layers arranged on top of one another, resulting in a deeper and higher level of abstraction. For the stacked LSTM implementation, we first built a sequential model, which is a linear stack of layers, and then added an LSTM layer with 8 units to the model by specifying the input dimensionality (22) for the first layer. The return sequences were true for the first layer. The dropout layer is then added, with a 0.1 dropout rate. Dropout is a regularization approach that randomly assigns a fraction of input units to zero during training to assist prevent overfitting. Another 8-unit LSTM layer is added, but this time the return sequences are false. Then, another dropout layer with a dropout rate of 0.1 is added. Finally, a fully connected layer (Dense) with 10 units is added, representing the number of classes or output categories in the classification problem. Because this is a multiclass classification problem, a softmax activation function is employed to convert the output of the preceding layer into probabilities appropriate for multiclass classification tasks. We created an EarlyStopping callback to monitor validation loss during training. The training procedure is terminated if the validation loss does not improve by a minimum of 1e-3 over 5 consecutive epochs (‘min_delta’). The ‘restore_best_weights’ parameter resets the model’s weights to those with the lowest validation loss. Overall, to counter the attack, we employed an LSTM model with numerous hidden LSTM layers, dropout layers for regularization, and an EarlyStopping callback to monitor the training process. Then we built the model with the loss function ‘categorical_crossentropy’, which is often used for multi-class classification issues. The optimizer is set to ‘adam’, an optimization method that changes the learning rate over time based on previous gradients. The metric used to evaluate the model is ‘accuracy’. The model was then trained using the training data and the corresponding encoded labels as input. The validation data and encoded labels are provided to assess the model’s performance during training. The ‘batch_size’ is set to 1000, which means that the model will update its weights after processing 1000 samples. The ‘epochs’ option is set to 50, which indicates how many times the model will iterate through the full training dataset. The ’callbacks’ argument is used to include the ’monitor’ callback, which can be an instance of ‘EarlyStopping’ that monitors validation loss and restores the best weights when training is terminated early. The object containing the training history, including the loss and accuracy values at each epoch, is used to display the accessible keys, which are usually ‘loss’, ‘accuracy’, ‘val_loss’, and ‘val_accuracy’. By examining the loss and accuracy matrices (Fig 5), as well as the confusion matrices (Fig 6), you may analyze the model’s performance throughout training. This data is important for additional analysis and visualization of the training process.

As previously stated, while implementing vanilla LSTM, a sequential model is first established, followed by one LSTM hidden layer with 22 units and an input dimensionality of 22 as the model’s first layer. The dropout layer is then added, with a 0.1 dropout rate. Eventually, a completely connected layer (Dense) with 10 units, representing the number of classes, is added to the model. The training process and hyperparameter settings remain the same as for stacked LSTM implementations.

## VI. Result analysis

The proposed DDoS detection system (Fig 1) is built using LSTM model which is one of the best deep learning algorithms capable of detecting and classifying DDoS attack. To get accurate results, the system is first learning the pattern or changed in attributes that is followed in happening of DDoS attack.

**Fig 7:**
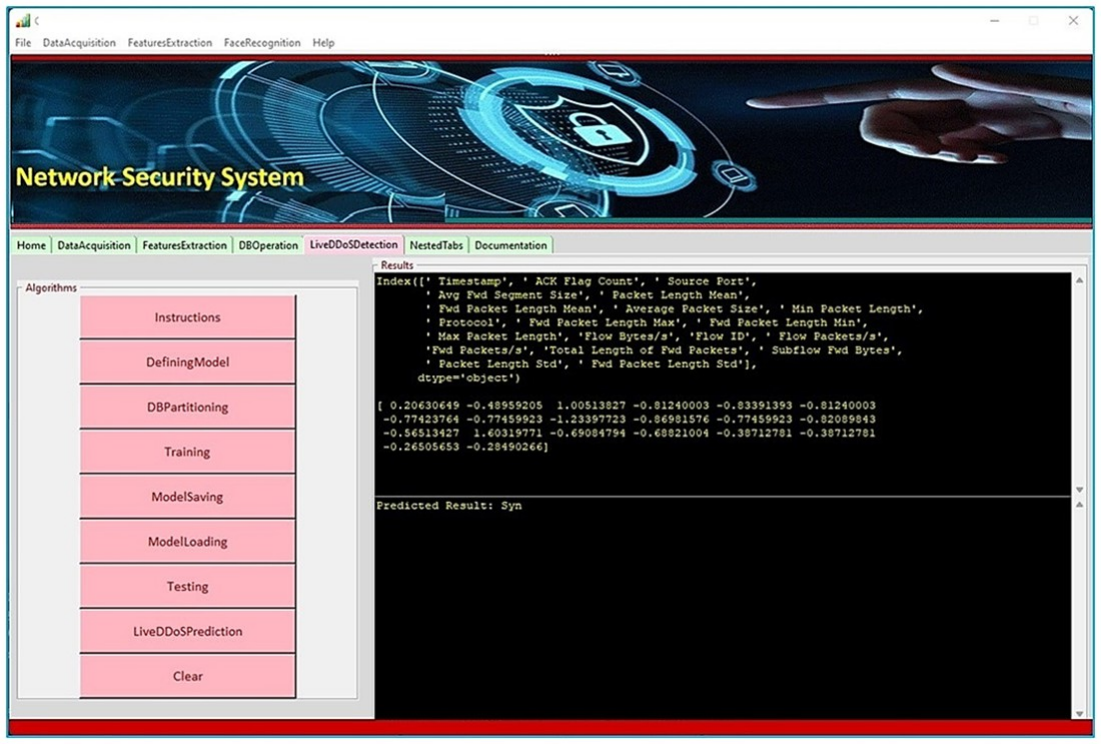
User interface that enables real-time DDoS detection.

The results of various of LSTM models are shown in Figs 5 and 6 as well as 2 and Table 3 with their corresponding features. The few findings deriving from the work as follows:

- The model is trained and tested with different varying rates with varying input data. The best result is achieved by 99.84% Vanilla LSTM model on the dataset of the test set respectively. Attack occurs in nanoseconds to milliseconds and affects the system. So, it can be concluded that this developed system is practically useful. It is also noticed that the computation time is very less compared to existing methods.
- In our project, 6332454 samples are used to train and validate the model, and 2713909 samples are used to test the model. It has been seen that the accuracy of the detection could be enhanced as the training dataset increases. A high-quality dataset is therefore required.
- Our system is able to detect the DDoS attack and it can also classify that which type of DDoS attack is this.
- It is found that there are only some (22) features which are changed during DDoS attack. The training of the dataset is making clear to generate a model that can check the alteration of these features.
- The findings of the experiment are statically evaluated and presented in Tables 2 and 3. It demonstrated that for the detection of the Distributed Denial of Service attacks the comparable classification accuracy and F1 score on the test set of our dataset.
- Our DDoS detection system is capable to produce result in real time as we have firstly achieved the results on benchmark dataset after that creating own real time dataset and it has given descent accuracy in detecting the attack on benchmark dataset and on real time dataset. Both the LSTM family models produce the results in comparable time. However, in real-time the inferences time varies greatly depending on the frequency of requests made or generated.
- Fig 7 shows that our DDoS detection system takes very less time in analyzing the change in features of network traffic of the system when DDoS attack is to be detected on the system, it determines the log files to analyze the features and checks those specific attributes that it has learnt by model training and detects the attack as the acquired changes occurs.
- Aside from various type of DDoS attack detection, our system also makes it possible to inform the user about the occurrence of DDoS attack by sending an alert message (Fig 7).
- Wireshark software is used in our work to acquire the information from network traffic in the form of the log files and after that CICflowmeter software is used to collect the features from the log files. These features are trained and tested by above explained method. And finally, our DDoS attack detection system is ready to be applied in real time.
- Eventually our system with enhanced accuracy will help to ensure the safety of a wide range networks by successful DDoS attack detection.

**Table 2:** Performance metrics of vanilla LSTM and stacked LSTM model based on confusion matrix.

Algorithm	Class	Precision	Recall	F1	Accuracy Score	Error Rate
Vanilla LSTM	Benign	0.99	0.99	0.99	99.84	0.16
	DrDoS_DNS	1.0	1.0	1.0		
	DrDoS_NTP	0.99	1.0	1.0		
	DrDoS_SNMP	1.0	1.0	1.0		
	LDAP	1.0	1.0	1.0		
	MSSQL	1.0	1.0	1.0		
	NetBIOS	1.0	1.0	1.0		
	Portmap	0.91	0.97	0.94		
	Syn	1.0	1.0	1.0		
	UDP	1.0	1.0	1.0		
Stacked LSTM	Benign	0.97	0.98	0.97	99.59	0.41
	DrDoS_DNS	1.0	1.0	1.0		
	DrDoS_NTP	0.99	1.0	0.99		
	DrDoS_SNMP	1.0	1.0	1.0		
	LDAP	1.0	1.0	1.0		
	MSSQL	1.0	1.0	1.0		
	NetBIOS	0.98	1.0	0.99		
	Portmap	0.93	0.61	0.74		
	Syn	1.0	1.0	1.0		
	UDP	1.0	1.0	1.0		

**Table 3:** Comparative analysis.

S. No.	Algorithm	Accuracy Score	Error Rate
**1**	**Vanilla LSTM**	**99.84%**	**0.16**
**2**	**Stacked LSTM**	**99.59%**	**0.41**
3	Deep Neural Network	99.48%	0.52
4	AdaBoost	52.06%	47.9
5	Random Forest	99.93%	0.07
6	GaussianNB	88.87%	11.13

## VI. Conclusions

Till now, Machine learning algorithms and Static methods were used, which contains limitations like Static approaches can perform good up to a time and every once in a while, there are new version of DDoS attacks came into picture that makes it difficult to detect via Static approaches and machine learning based approaches. In Our proposed approach, we have used deep learning (DL) models because DL models are rarely used to detect DDoS attack and able to handle a large amount of dataset that is a key-factor in attaining maximum accuracy while machine learning and other static approaches are not proficient in this context. By extracting the important features, after training the model we have successfully developed deep learning based Long Short-Term Memory (LSTM) like Vanilla LSTM and Stacked LSTM, and Artificial Neural Network (ANN), also AdaBoost, Random Forest, GaussianNB models to classify the CICDDoS2019 dataset and achieved ~99.5 accuracy rate that is significantly better than other available methods. The difference between the results obtained from vanilla LSTM, stacked LSTM, ANN, and other models detail that Vanila LSTM along with Random Forest models should be used to detect DDoS attack to perceive maximum accuracy and lower error rate. In future we will apply these techniques on multiple realistic datasets those will be created in different-different topologies along with this it will be suitable to develop a real-time scenario.
